# Community responses to communication campaigns for influenza A (H1N1): a focus group study

**DOI:** 10.1186/1471-2458-12-205

**Published:** 2012-03-19

**Authors:** Lesley Gray, Carol MacDonald, Brenda Mackie, Douglas Paton, David Johnston, Michael G Baker

**Affiliations:** 1 Dept of Primary Health Care and General Practice, University of Otago, Wellington, New Zealand; 2 F6 Mahunga Drive, Masterton, New Zealand; 3 School of Social and Political Sciences, Canterbury University, Christchurch, New Zealand; 4 School of Psychology, University of Tasmania, Launceston, Australia; 5 Joint Centre for Disaster Research, GNS Science/Massey University, Lower Hutt, New Zealand; 6 Department of Public Health, University of Otago, Wellington, New Zealand

## Abstract

**Background:**

This research was a part of a contestable rapid response initiative launched by the Health Research Council of New Zealand and the Ministry of Health in response to the 2009 influenza A pandemic. The aim was to provide health authorities in New Zealand with evidence-based practical information to guide the development and delivery of effective health messages for H1N1 and other health campaigns. This study contributed to the initiative by providing qualitative data about community responses to key health messages in the 2009 and 2010 H1N1 campaigns, the impact of messages on behavioural change and the differential impact on vulnerable groups in New Zealand.

**Methods:**

Qualitative data were collected on community responses to key health messages in the 2009 and 2010 Ministry of Health H1N1 campaigns, the impact of messages on behaviour and the differential impact on vulnerable groups. Eight focus groups were held in the winter of 2010 with 80 participants from groups identified by the Ministry of Health as vulnerable to the H1N1 virus, such as people with chronic health conditions, pregnant women, children, Pacific Peoples and Māori. Because this study was part of a rapid response initiative, focus groups were selected as the most efficient means of data collection in the time available. For Māori, focus group discussion (hui) is a culturally appropriate methodology.

**Results:**

Thematic analysis of data identified four major themes: personal and community risk, building community strategies, responsibility and information sources. People wanted messages about specific actions that they could take to protect themselves and their families and to mitigate any consequences. They wanted transparent and factual communication where both good and bad news is conveyed by people who they could trust.

**Conclusions:**

The responses from all groups endorsed the need for community based risk management including information dissemination. Engaging with communities will be essential to facilitate preparedness and build community resilience to future pandemic events. This research provides an illustration of the complexities of how people understand and respond to health messages related to the H1N1 pandemic. The importance of the differences identified in the analysis is not the differences per se but highlight problems with a "one size fits all" pandemic warning strategy.

## Background

During an influenza pandemic the lack of experience of dealing with such hazards increases public reliance on information from government, public health agencies, employers, the community and the media. A challenge for government and health agencies is sustaining public awareness and alertness over a protracted period [1,2]. Responding to the challenges posed by managing the risk from relatively unknown health hazards requires recognition of the fact that promoting sustained action involves not only getting the information right but also ensuring that it is communicated in ways that accommodate diversity in community characteristics, needs and expectations. The lack of experience also calls for research to provide the evidence base health authorities need to respond effectively in circumstances (e.g. rapid onset of disease outbreak) that precludes learning in situ.

There is evidence that greater levels of perceived susceptibility to and perceived severity of the disease and greater belief in the effectiveness of recommended preventative and avoidant measures are important predictors of behavior [3-5]. It is important to accommodate the fact that disbelief in the effectiveness of measures can result in people failing to act and developing distrust of sources of information [6]. Previous New Zealand research identified a general belief that local agencies will manage a pandemic well and that New Zealand is a relatively safe place to be in the event of a pandemic. However, there was also a lack of trust in information providers as well doubt that the health system would cope [4]. Trust is a crucial component of effective risk communication when people face uncertainty and levels of trust are correlated with people's perceptions of the integrity of information. Lack of trust can be exacerbated by scepticism about the veracity of health risk warnings and the view that media are sensationalist and untrustworthy [7,8]. Being uncertain about an outbreak, and thinking it, and its consequences had been exaggerated have been associated with a lower likelihood of behavioural change [5]. Increasing trust is a function of the degree to which agencies engage with and empower communities [6].

Lack of trust in authorities may also affect how people process and interpret health messages and advice, increase concerns and interfere with the way that the risk messages are interpreted and acted on [9]. Transparency and honest communication where both good and bad news is conveyed can empower the public to make their own decisions [1]. The public are more likely to take appropriate action and accept the recommendations if they have been involved in the decision-making process, and the quality of the relationship between authorities and the community has a direct effect on the uptake of risk messages, and trust in the message providers [2].

The primary objective of this study was to provide health authorities with evidence-based practical information to guide the development and delivery of key health messages for H1N1 and other health campaigns. The study focused on community responses to key health messages in the 2009 and 2010 H1N1 campaigns.

## Methods

The study was part of a rapid response initiative; therefore focus groups were selected as the most efficient means of data collection in the time available. Eight semi-structured focus groups were recruited between May and July 2010 (the New Zealand winter season) comprising 7 to 13 participants each and lasting approximately 1 hour. Separate focus groups were conducted for each of the target groups with a total of 80 participants representative of five target populations groups identified in consultation with Ministry of Health staff: Māori, Pacific Peoples, children (or parents of children), general population, and vulnerable people with chronic conditions (defined as those who are eligible for subsidised vaccinations, such as pregnant women, those with diabetes, using asthma inhalers, with heart disease or kidney problems. Participant characteristics are summarised in Table 1.

**Table 1:** Participant characteristics

	N (%)
**Gender**	
Male	15(19)
Female	65(81)
**Ethnicity**	
NZ European	16(20)
Māori	28(35)
Pacific	22(28)
Other	7(9)
Not stated	7(9)
**Age**	
18-24	5(6)
25-34	14(18)
35-44	11(14)
45-54	21(26)
55-64	14(18)
65+	14(18)
Ns	1(1)
**In paid employment**	
No	22(28)
Yes	33(41)
Not stated	25(31)
**Health conditions**	
Yes	36(45)
No	31(39)
Not Stated	13(16)
**Focus Group Category**	
General Population: with children	G1
General Population: chronic conditions	G2
General Population: mixed	G3
Pacific Peoples: chronic conditions and pregnant/very young children	P1
Pacific Peoples: non-chronic.	P2
Pacific Peoples: chronic conditions	P3
Māori: Kaumātua	M1
Māori: Tamariki Ora Mothers (young Māori mothers)	M2

Purposive sampling methods were used to ensure the sample met the criteria specified by the Ministry of Health.

The period of data collection was selected to coincide with the period in which people are most aware of flu and the need for preventive and protective actions. Participants were recruited using posters in a community library and through contacts within key agencies (e.g. university research centres, Pacific health services, district health boards, and the Ministry of the Social Development). Staff of the Research Centre for Māori Health and Development,

Massey University were responsible for the Māori components of the research to ensure a culturally appropriate methodology given the time restraints of the study. In contrast to New Zealanders of European descent, Māori possess different cultural characteristics. For example, based on cultural dimensions [10] Māori score higher on dimensions such as individualism-collectivism, power distance and uncertainty avoidance compared with their counterparts. Because these differences have significant implications for social influences on behaviour and for the nature of the relationships that exist between community members and health authorities, it was essential to ensure that data were collected in culturally sensitive and competent ways. Hence Māori were treated differently from the perspective of the data collection approach adopted. This is consistent with effective cross cultural research methods [11].

All focus group sessions (except Māori groups) were recorded and independently, professionally transcribed. Transcriptions were mainly verbatim, with verbal padding and hesitations omitted. Apart from the facilitators, specific individuals were not identified in the transcripts or any subsequent reports. The extracts used to illustrate the content of each theme are identified by codes which correspond to the focus group transcripts from which they were taken (Table 1).

The analysis of the focus group data (excluding Māori groups) was undertaken by a single researcher who was neither present at the focus groups nor had read any preliminary findings. This work was verified by the focus group facilitators to ensure that any "contextual richness" had not been missed in the data. Thematic analysis was used to identify themes and concepts across the entire data set (6 transcripts) to "identify repeated patterns of meaning" [12]. The process involved working through the six phases of thematic analyses as identified by Braun and Clarke [12].

This study was approved by the Massey University Human Ethics Committee: Southern A (ref 10/32, 21 May 2010). Written informed consent was obtained from all participants.

## Results and discussion

The final coding scheme consisted of 17 themes which were grouped into four main categories: risk, building community understanding, responsiveness and information preferences. Transcript extracts were selected on the basis of their relevance to the theme under discussion and all identifying information has been removed.

### National preparedness and risk

People's perception of risk is a product of the perceived likelihood of a pandemic and its perceived consequences. The analysis identified that people made judgements about both likelihood and consequences. Understanding these inputs helps tailor risk communication strategies. Previous research identified a general belief that New Zealand, as a result of factors such as geographic isolation, reduce the likelihood of a pandemic and thus increase a belief that New Zealand is a relatively safe place to be in the event of a pandemic [4]. Perceived risk was also attenuated by a belief that border control agencies will prevent pandemic flu entering New Zealand, with this possibly resulting in people transferring responsibility for preparing from themselves to border control agencies [6].

"It's pretty much a one-border control place. That's why that's better. We're lucky, because we've got only that one contact point coming in." (G3)

Other participants were aware that our perceived geographical isolation does not in fact translate to a decrease in risk. In fact H1N1 arrived in New Zealand early in the global pandemic of 2009 with students returning from a school trip to Mexico and the USA.

"Well we think that we're different because we're far away. But actually, if you think of how people travelled here, it's the biggest factor for it always, because everyone who comes here comes in an aeroplane, pretty much. And they come from everywhere." (G3)

### Concern and risk acceptance

Discussion reflected variable levels of awareness of and reaction to pandemics. Many regarded the 2009 H1N1 pandemic as an overreaction that was not taken very seriously by many people. The following extract illustrates the concept of "normalisation bias" in which people extrapolate from the current experiences (H1N1) to define what a future pandemic would look like and therefore underestimate their future risk which makes them less respective to current health risk messages [6].

"I thought it was scaremongering, personally. I thought the way it was handled was quite interesting, and I think it also made a lot of people very paranoid about things that sometimes people don't need to be paranoid about." (G3)

For some participants it was the use of term "pandemic" that gave rise to the sense of overreaction by suggesting something more serious than what actually occurred.

"It became reasonably clear reasonably quickly last time that hundreds and thousands and millions weren't dying. Even when they kept on sort of saying things were happening, and then you saw the numbers, it just didn't add up." (G1)

There is evidence that higher levels of general anxiety and perceptions of high risk severity are related to a greater likelihood of carrying out preventive and avoidant protective behaviours [3,5]. However, it is important to note that this relationship is only present when people with high risk perceptions also know what to do to manage their risk. If people do not know what to do, or question the veracity of the advice offered, they are more likely to respond by denying the risk or transferring it to others [13]. Whilst most of the participants in the general population groups regarded swine flu as "bit of a joke, really" others reported feelings of anxiety, fear or panic. In particular, reports of flu-related deaths gave rise to more serious concern.

For those who felt that geographical remoteness provided some form of protection, it was not until cases were reported within New Zealand that the threat became more real. This shift in thinking is important as the perceived relevancy and immediacy of risk affects the decision to act or not to act on information [4,8].

"Because you kind of go, well, we're just little old New Zealand, where nothing happens. You know, we'll be right. And it wasn't until you actually heard that people in New Zealand had brought it back from overseas. And that's when you really does go "Ooh! Alright." (G3)

Concern about news reports from overseas also related to the reliability of the information in terms of both its trustworthiness and relevance to "us".

'The other thing, people are a bit cynical about the whole thing. Because there are these people dying in Mexico, you think, well, there are people sick in hospital. You know, I've got no idea what the health system's like there." (G2)

Such comments reflect a belief "it won't happen to me because - it happens to others". Through the process of "othering", individuals focus on differences in others, effectively creating a separation between "us" and "them" [14]. By projecting the risk of infection and death onto "them" the sense of powerlessness and vulnerability is reduced for "us" [15].

Cynicism about the veracity of media reports was not limited to news from overseas. Indeed there was a strong feeling that the New Zealand media had a central role in the "overhyping" of the 2009 and 2010 pandemic risk. Media are credited with amplifying the risk perceptions in such a way that risk communicated may not be an accurate reflection of the true risk [7].

"I think the news media have a lot to blame, because they want to make news. They sensationalise stuff. That's not helpful." (G2)

Public distrust in journalists and the sensationalising of health related stories can also be a hindrance to taking the risk seriously and of undertaking precautionary measures [5]. A belief that risk has been exaggerated is associated with an increased sense of helplessness and frustration and a reduction in the likelihood that people will prepare in the short-term [5,7,16].

### Contextualisation and saliency

In an attempt to understand potential risks people situate risk-knowledge in historical and local contexts [17]. This can be seen in the focus group discussions in which participants were more concerned about other risks, including illnesses such as heart disease, cancer, meningitis, and respiratory disease.

Much of the discussion in the general population groups focused on their reassuring themselves that the pandemic was indeed an overreaction and a "media beat-up" as they believe that this had happened before with other events. In this condition, people preferentially seek information that reinforces this belief and this may reduce the likelihood of their attending to public information. Maintaining a sceptical and cynical perspective is a means of "distancing" themselves from the threat.

"Like the bird flu that was... that, I think, killed some... a few people in Canada. And people said it was going to be the next big flu, and it wasn't." (G2)

### Risk perception

There is evidence that individual perceptions of risk are important determinants in undertaking preventive, and/or protective behaviours for events such as pandemics as long as people know how to manage their risk [3,5,12,21]. Some participants appeared to have a sense of "personal invulnerability" based on their history of rarely having experienced flu's or colds. The comments were also indicative of the phenomenon of unrealistic optimism. This means that while people accept the existence of a risk to the community in general, they see themselves as less vulnerable or more capable than others. This bias results in their transferring risk from themselves to others and seeing risk communications as applying to others rather than to themselves [6]. This bias represents a significant constraint on the effectiveness of risk communication. However, encouraging active discussion of pandemic issues in community groups can reduce its influence.

"I've been fortunate enough to... I don't know that I've even actually ever had the flu, even ordinary flu, in my life... So I seem to have a bit of a natural immunity to it, luckily enough, so... yeah." (G2)

In contrast there were others who expressed a heightened sense of risk due to their personal circumstances and health history. Assessing their personal risk in this way supports the view that people's understanding of risk is developed not only through cultural and sub-cultural membership, but also through personal experience [17]. This highlights the importance of risk communication encouraging people to personalize information.

"I've always had a problem too, because my partner's a primary school teacher by trade. She'd bring the things home. ...There was this flu going round, and I'd be getting it". (G2)

While all groups were aware of the concept of "high risk" groups the relationship between knowledge of "high risk factors" and individual perceptions of risk was less clear. Although some participants identified themselves as "high risk" they appeared to be uncertain about what this meant for them. Others used "othering" to minimise their own perception of risk by differentiating themselves from those who they identified as "high risk". This social construction of boundaries of "self" and "other" and their relationship to boundaries of "safety" and "danger" are particularly relevant to understanding notions of health and disease [18].

However, the self/other divide can be used to facilitate preparing [13]. By considering whether something can be done to assist those more vulnerable, people are more likely to also consider what they can do for themselves.

### Key health messages

Recall of key health messages was varied, however most participants were aware of hygiene self-efficacy measures such as hand washing, sanitiser use, covering of coughs and sneezes, and staying at home. This was particularly strong in one of the Pacific Peoples groups and for many appeared to be translated into action.

"Mainly they tell you to wash your hands....Cover your mouth when you cough.......And don't share hankies, they say, yeah." (P2)

There was some recall of the 2009 posters and the messages which they contained. Although some who recalled the posters expressed concern that they were inaccurate and overused. There was generally less recall of the 2009 television advertisements, and even less recall of the messages they were conveying. Overall, participants did not feel that they were better prepared or had changed their behaviour as a result of the information which they recalled from 2009. Where lessons had been learnt from the previous year, these were mostly related to improved personal hygiene measures.

Awareness of the 2010 campaign was scant and what was recalled tended to be from commercial advertising, for example, from private commercial companies promoting flu vaccinations and household hygiene products.

### Information sources

Participants reported receiving pandemic information from a variety of media sources including newspapers, TV, radio and the internet. However the primary source of information for participants was their workplace and/or community. This differs from previous research in which Google was listed as a primary source of information [8] and television was the preferred means of receiving information during a pandemic [19].

The general population groups tended to report workplace as a key source of information, including workplace intranets. In contrast there was general agreement in the Pacific Peoples groups that their primary sources of information were the community. This included social networks and family, regular forums and meetings, church groups and health centres. This supports the view that when faced with uncertainty, people turn to others to reduce their uncertainty and guide their preparation; often these are family and friends, but also health agencies with whom they have a direct relationship [2]. This highlights the importance of both recognising the existence of diverse communities that facilitate developing understanding and engaging with these communities to ensure that information can be tailored to meet the needs, goals and expectations of each group.

"And even in the church. Because one thing in the church is, our people fear God, and they always go church no matter what. And the pastor is one of the key people that talks into the community." (P2)

The 2010 campaign had largely gone unnoticed by Māori tribal elders (kaumātua). They did not believe that information was readily accessible, no one had seen articles in the local press regarding H1N1 and pamphlets and posters "were not freely available". This view was mirrored in the young Māori mothers (Tamariki Ora) focus group.

The kaumātua group felt that information is best disseminated to places of work, school and family and to Māori health providers to ensure coverage of the Māori population. It appeared that the messages in the media did not make an impact with Tamariki Ora mothers.

### Community strategies

Previous research has shown that community participation and trust in emergency management agencies played significant roles in increasing community preparedness, willingness to take responsibility for own safety, risk acceptance and satisfaction with communication [2]. Public are more likely to take appropriate action and accept the recommended actions if they have been engaged in all aspects of the risk management and decision-making processes through mechanisms such as focus groups or forums in ways that empower people to take action [6,20].

Kaumātua were of the view that the 2007/08 campaign had been successful because the District Health Board had come out into the community and involved them in planning or providing information, but "it had not been effectively followed up on". A number of other comments also highlighted the importance of engaging with communities and disseminating information through community mechanisms.

"Civil Defence needs to be proactive, and actually make sure that they've got the right community people, and the right community organisations on the board." (G3)

With respect to workplace pandemic or disaster response plans, most participants had little or no knowledge of these. Some were aware of general plans or the existence of emergency supplies at work. There was a general belief in one group that emergency preparedness was seen as an "individual responsibility" by their employers. The apparent lack of clearly-articulated workplace response strategies is consistent with previous research [20].

### Preparedness

As with previous research [2,20,21] few participants had stocked up on emergency supplies or prepared for the pandemic any other way. Some individuals had stocked up on food and essential supplies and/or had a family disaster plan.

"I've certainly thought about how would we get food, or how much food did we have in the house, if we were to get quarantined." (G1)

While some participants reported that they had already prepared emergency boxes, others were yet to act on their intentions to do so; it was on the list of things to do. Participants were aware of advice to stock-up on pharmaceutical products but a number found the array of over the counter products available very confusing.

Perceived or actual economic impact influences psychological and behavioural responses [22]. Many participants expressed concern that the cost of emergency kits could be a barrier for low income families and that some sort of financial assistance should be available, particularly as they may believe that the costs of acting will only incur a benefit in the event of a pandemic and this may not occur until some future date.

"And the thing is that it would affect... I mean, imagine someone on a really base-level income... isn't going to have a preparedness kit. So they're the ones that are going to suffer, through in some ways, no fault of their own." (G2)

### Vaccinations

Attitudes towards having the flu vaccination varied greatly as did uptake. Discussion concerned H1N1, H5N1, pandemic flu and seasonal influenza. There were those who routinely have them and those who "don't do flu shots". Uptake rates ranged from none in the Tamariki Ora mothers group to over 90% of those present in the kaumātua group.

For some participants the decision whether or not to have the vaccination was related to their perception of risk.

"Yes. I normally do have it. But I insisted on having it early, even though the GP was only giving it to people who were in vulnerable groups, because of the fact that I was travelling ..." (G1)

There were others who, having identified themselves as being in an "at risk" group, still chose not to be vaccinated. For these and others it appeared to be a balancing act between their perceived risk of influenza against the perceived risks associated with the vaccine itself. The "costs", psychological and health "benefits", and expected outcome are all important issues that influence people when making a decision about whether to act on advice about a pandemic [2].

"I'm kind of stuck on that one. I've certainly thought more about it this year, as to whether I should just take the risk; but I'm not going to. I'm still not having one." (G1)

Several of the Tamariki Ora mothers were apprehensive and confused about the flu injection and wanted more information about "immunisation for their children".

"Will the current flu injection help us to stay immune for several years?" (M2)

Whilst some participants elected not to have the vaccination even if it was free, for others cost was a significant issue. There was also a degree of cynicism about the vaccination and flu treatment amongst those that reported not having had one. In particular cynicism was expressed with respect to Tamiflu^®^.

"The whole Tamiflu... again, it makes me cynical, you know. Somebody was making a heck of a lot of money out of that, you know?" (G2)

The reluctance to be vaccinated and the cynicism illustrated by these extracts is consistent with research showing that decisions to engage in preventive and avoidant behaviours is influenced by attitudes towards public health interventions [9] including having confidence in the efficacy of the behaviour [3]. It is worth noting that the latter beliefs influence the level of trust in health agencies and that specifically advising people (about all preparedness measures and not just antivirals) about why specific preparations are required increases the likelihood of adoption and helps maintain trust in health agency sources of information [12].

### Staying home and social distancing

Participants had heard the social isolation/distancing message.

"If you're sick, go home. And if you're sick and you're at home, stay there....That's right. I'm quite vocal about that anyway. I hate seeing people sick around me." (G1 with agreement from several other participants)

Although participants recognised that isolation is an important response strategy, the economic pressures to go to work instead of staying at home was a major concern.

"That's a big push, yeah, that's a big reason why people still go out, even though they know they have a cough. ...Yeah, that pushes me (into employment). ......You send your kids to school sick. .........Yeah, because you haven't the time, yeah." (P2)

This is consistent with previous research showing that perceived or actual economic impact is a major factor in decisions around avoiding the flu [9,19,21,22]. These issues can be compounded by not preparing, being unaware of workplace policies and plans (e.g., policies about wage payments if people are advised to stay home).

Kaumātua believed should a person develop flu-like symptoms they should isolate themselves from other members of the community, however they were concerned that when individuals are isolated they may not be contacted by members of the community or whānau. Concerns were also expressed by this group about observing specific cultural practices and greeting protocols, reflecting the role that cultural and sub-cultural membership has in people's understanding of risk [17].

Participants wanted guidelines about who should stay home and when, and they wanted backing from their employers with respect to this issue. Participants felt that they had been given contradictory information about when to stay home and when to go to work or school which left them feeling uncertain about what to do. Consistency of advice is a significant important factor in communications from key agencies [3].

### Information preferences

"Knowing the difference" between swine flu and other flu emerged as a significant issue. This reflects previous reports of a strong desire from the public for symptom details about influenza [19] and the finding that public information about signs and symptoms are beneficial to public understanding of a pandemic [8]. Participants across all groups wanted to know about the specific swine flu symptoms which they could use to identify it and protect themselves from possible infection.

"I mean, the big thing is what are the symptoms, particularly what are the unique symptoms to whatever the pandemic is, that differentiates it from regular flu, or a cold? And how infectious is it, and what's the mechanism of infection" (G1)

There is a view that surveillance combined with good scientific information and operational research is crucial in limiting the spread of H1N1 [23]. A number of participants were concerned about testing and monitoring. They felt that the Ministry should not have "cut off monitoring quite so early" and that the failure to test for the H1N1 after health services became inundated with patients undermined the advice that the Ministry of Health was giving.

### Facts

In addition to the desire for symptom details many participants wanted concrete facts, such as how many people were diagnosed with or dying from swine flu. Some participants wanted to know percentages; others preferred the information to be given as numbers because they found percentages confusing. For many participants it was not necessarily numbers they wanted but information that helped them judge the "seriousness" of the pandemic and their level of personal risk.

There was general agreement in a Pacific Peoples group that simplicity in the framing of messages was important. This aligns with the contention people can absorb only a small amount of information at a time and have difficulty understanding some kinds of information. Risk communication should take this into account and identify the most critical facts [24].

### Trust and honesty

It is apparent from previous research that trust in authorities and satisfaction with communications received are associated with compliance of preventive, avoidant, and management behaviours [2,3,22]. People want the truth, even if it is worrisome, so honesty is crucial [24] even if that meant being told "we don't know, at this stage".

"Give it to us the way it is. Because I'm sure adults are capable of dealing with that information, and then, you know, making their own choices later of how they deal with the information, but to actually be given that information, without any drama, and yet not being, you know, pushed under the rug somewhere." (P3)

While some participants expressed confidence in the organisations providing information, others felt that they were not being given all the facts and that this affected their ability to make informed decisions. Trust in the information given is important because it affects the perceived credibility of risk assessments from authorities which in turn can influence response behaviours [3]. It is important to note that trust can be easily lost if people believe that agencies are not acting in their interests or do not provide information that meets their needs and once lost, trust is difficult to regain [6].

"Well, I'm always dubious about the... particularly the death rates that... that was not real... from what I understand, a lot of people that died had pre-existing conditions." (G2)

Transparency and honest communication where both good and bad news is conveyed empowers the public to make their own decision [1] and that openness of government communication and acknowledging uncertainty is important for fostering trust [3].

Consistency of advice also appears to be an important factor in communications from key agencies [3]. Participants provided a number of examples where conflicting information or advice led to a feeling of confusion and frustration and loss of trust in key sources.

"...at the beginning of the swine flu, the communication breakdown between the hospital and the local GP services. Because people were coming to the GPs, and they were referring them to the hospital, and then they were... (saying "No, we only take emergencies"), there's a lot of confusion." (P1)

Dissatisfaction with health providers was not limited to poor inter-agency communication. Just getting an appointment with a GP was difficult for some and others had concerns about how they were treated and the advice which they were given. The issue of financial impact is also relevant to seeking medical assistance with a number of participants reporting that the high cost of a doctor's appointment was definitely a deterrent to seeking treatment. The issue of trust is illustrated by this extract from a woman who believes she was misdiagnosed in the emergency department putting vulnerable family members at risk.

"I don't think she knew what I had. I just think she wanted to go tick, "Goodbye, here's the antibiotic." ... and I believed her. And that's how naive I was. I should have gone again, but I thought, "Oh no, she's got the ticket. She's got the certificate that says 'doctor'." I trusted her. ... when I came here further, I had the swine flu, I just wanted to get out and go back up there and find that little lady, and taking her and her certificate, and bang her up the side of the head and say, "You could have taken out my granddaughter, my daughter, and my father, because you were in too much of a hurry to go tick, tick, tick." (P2)

### Practical information

Focus group participants expressed a desire for practical information to guide their responses to the influenza threat. Such views are consistent with previous reports of research participants preferring risk messages that empower with information about actions that could reduce risk and/or mitigate consequences [4,5,12]. People want to know how to protect themselves and their families during an influenza pandemic and the ability to act provides a sense of relief that "they could do something" [8]. It has been reported that participants who received little or no information about protective actions they could take, expressed helplessness and frustration [8],

### Targeted messages

Language preference has been shown to be an important factor in satisfaction with risk communications [4]. Participants in the Pacific Peoples groups stated strongly that messages should be communicated in an appropriate language.

"Sometimes our older folk, they don't understand English. They have to be in original languages. The diversity of the Pacific - I mean, for some of our older people, because it's their cultural background, and it's so hard for them to understand." (P2)

The acceptance of public health messages can be affected by factors such as socio-cultural behaviours, gender roles, generational differences, religious beliefs and language preferences [9]. The following extract also supports the argument that emotions can also cloud people's decision making, so communicators must treat audiences respectfully [24]. In order to accommodate these cultural and demographic factors it is important to work through communities.

"Sometimes I don't think it's just not only their language....it's the way you use your language. You speak too fast, your English words are beyond me. And they're not dumb, the people. .... Sometimes it's just the way you... your tone. If you talk to them like they're dumb, well they'll just...They'll shut up." (P2)

The following comment from a kaumātua also supports the argument that key health information and advice must take into account factors such as socio-cultural behaviours, spiritual beliefs and language preferences. In the event of an outbreak or pandemic, it would be difficult for kaumātua not to observe the protocols that are very much part of their traditional practices.

If there was a outbreak we would have been ok, we were concerned at the lack of knowledge of tikanga Māori and it is not usual to have to stay away from marae or to stay at home, the thought of mass burials in a pandemic was culturally irresponsible." (M1)

### Timing and frequency of messages

Discussions around preferences for the timing and frequency of key health messages were contradictory. On one hand participants, especially those in one Pacific Peoples group expressed a desire to be given early information through frequent messages. Other participants however, felt that they were being "bombarded" with too much information too soon.

Participants wanted to be warned about potential risk well in advance to allow time for them to prepare. This supports the view that occasional media reports are insufficient to adequately inform individuals about pandemic preparedness, and interventions are needed before a pandemic occurs to improve public awareness, promote effective coping responses and help in the successful implementation of plans [25].

In contrast others thought it would be better if information was given much closer to the actual event. This is consistent with previous research that found participants preferred "just in time" delivery of information to avoid having to think about pandemic influenza unless they had to or unless a pandemic was imminent [8]. "Just-in-time" messaging that included technical terms, risks, health benefits and protective actions has been shown to help align public perception with realistic assessments of pandemic threat [24]. Both of these perspectives reflect problems with people's risk beliefs. Those who desire advance warnings may be unaware that a pandemic could be in New Zealand very quickly and possibly before its existence is formally identified. They may not have the time they expect to prepare. Those who adopt a "just in time" approach may overestimate their capacity to prepare in a short time frame (e.g., food supplies in supermarkets being rapidly depleted and not restocked).

"I don't know we should say anything unless there's a clear danger, because otherwise you just get used to it. I mean you ignore it." (G1)

The extract above illustrates the serious problem that arises from warning fatigue. Too many early warnings can result in cynicism, disengagement and a decline in trust [26]. Warning fatigue is more likely to result from potential threats with a 'long-lead-time'- like pandemics, where there are many warnings in the absence of the actual threat. People "get sick of hearing it" and are likely to "switch off" and ignore future warnings.

"But I think part of it is, that we've had so many health scares in the past decade. Like SARS, and different types of flu, and it's all this big media hype thing....and then it just kind of disappears." (G3)

### Effective communications

Focus group discussion about other information campaigns highlighted differences between what participants considered to be effective communications and from who they wanted to hear important messages. Some advertisements were seen as very effective because they were "straight-up, to the point" and included people to whom the participants could relate.

"Because they're using the Pacific People, and the different languages. Like after the Cook Island one, they have a Cook Island lady saying, ("Don't have hesitation")." (P2)

The perceived success of other campaigns was associated with the person fronting that campaign as much as the quality of the presentation.

"Going back to those first John Kirwan (sporting celebrity) ads, they were just really nicely shot, with someone that... most New Zealand men would respect John Kirwan. He was the ideal person." (G1)

Such comments confirm previous research that shows that people are more likely to act when information comes from within their own community; community leaders are highly credible sources of information and people would prefer them to be trained in issues of pandemic risk management [27].

The front person was an important factor in presenting important messages. There was some interesting debate about who could "be trusted" and who was "believable". Some participants felt that important messages should come from medical professionals or official agencies such as the Ministry of Health, District Health Board or WHO. This view highlights the importance of credibility which is regarded in the literature as a critical element in effective risk management and communication [28,29]. Communicators who are also scientists and perceived as being impartial and knowledgeable are more likely to be regarded as credible [28].

"Well, the Ministry of Health, ideally, should front it, because you know, they're the national organisation." (G3)

Others argued that the front person should be a role model, or someone recognisable to the public at large.

"...I think it might stick in your mind a lot more if you'd got someone that looks familiar to you telling you the information. And I think that we do actually trust someone that maybe is a household name, more than someone that they have no idea who they are". (P1)

As some participants pointed out, it may be that a variety of communicators may be best. Indeed there is support in the literature for employing a multidisciplinary approach to risk communication with input from a range of experts [1,24].

### Limitations of this study

Any conclusions drawn from this study should be considered tentative as the findings cannot be generalised to the population at large. It is not known whether the individuals who chose to participate differed from those who were eligible but chose not to participate. Whilst this study intentionally involved participants with diverse cultural and ethnic backgrounds, and included individuals from vulnerable groups, the sample does not permit conclusions regarding the effect of socio-demographic factors such as age or gender. Further research is needed to explore the complexities involved in the way in which the framing of risk messages impacts on people's perception of risk and subsequent preparedness and response behaviours.

## Conclusions

The results of this study highlight the problem with a "one size fits all" pandemic warning strategy that risks antagonising and distancing communities and thereby reducing trust in agencies and the likelihood that advice will be followed. Agencies must acknowledge that the public are diverse and need to be involved in the development and management of pandemic response initiatives appropriate for different communities and sensitive to existing cultural and/or spiritual practices.

Pacific Peoples and Māori focus groups identified community institutions with established mechanisms which could provide useful vehicles for the dissemination of information and engaging with the community. With a community engagement perspective the role of the health agencies would be that of consultant to the community or a change agent rather than trying to disseminate directly to the public in a top-down approach. For all segments of the population, the effectiveness of risk communication can be increased by using community engagement and empowerment principles that help tailor information to the needs and expectations of diverse groups.

Pandemic management strategies need to be seen in relation to the wider context of overall societal emergency preparedness. Preparedness for specific events, such as pandemics, might be better situated within more general risk campaigns rather than as stand alone approaches. Such a collaborative approach would also help reduce the sense that people have of being bombarded with information from multiple sources with frequently conflicting messages.

Information alone is insufficient to motivate people to prepare. The way in which information is presented or conveyed is an important factor in determining an individual's response. People wanted messages about specific actions that they could take to protect themselves and their families and to mitigate any consequences. They wanted transparent and honest communication where both good and bad news is conveyed. There was a desire across all groups for clear and specific information, such as infection and/or death rates and defining symptoms. This reflects a failure to distinguish between the pandemic and its consequences and highlights the importance of doing so for risk communication.

## Competing interests

The authors declare that they have no competing interests.

## Authors' contributions

LG contributed to the formation of the study, conducted focus groups, and contributed significantly to the manuscript. CM contributed to the formation of the study, performed the analyses and contributed significantly to the manuscript. BM reviewed the literature, conducted focus groups, and contributed to the manuscript. DJ initiated and contributed to the study. DP contributed to the study and the manuscript. MB contributed to the study. All authors read and approved the final manuscript.

## Pre-publication history

The pre-publication history for this paper can be accessed here:

http://www.biomedcentral.com/1471-2458/12/205/prepub
